# Hybrid Carbon Nitride/Cobalt Phthalocyanine Nanocomposites for Efficient Photocatalytic Hydrogen Generation

**DOI:** 10.1021/acsaem.4c03257

**Published:** 2025-04-04

**Authors:** Lakshman Sundar Arumugam, Javier E. Durantini, Jorge Follana-Berná, Frederik Schiller, Ane Etxebarria, Lorenzo Forzanini, Sara Barja, Ángela Sastre-Santos, Sixto Giménez

**Affiliations:** † Institute of Advanced Materials, 16748 Universitat Jaume I, Avinguda de Vicent Sos Baynat, s/n, Castelló de la Plana 12006, Spain; ‡ Área de Química Orgánica, Instituto de Bioingeniería, Universidad Miguel Hernández de Elche, Elche 03202, Spain; § Centro de Física de Materiales CFM (UPV/EHU-CSIC), 202635 University of the Basque Country UPV/EHU, San Sebastián 20018, Spain; ∥ Donostia International Physics Center DIPC, San Sebastián 20018, Spain; ⊥ Department of Polymers and Advanced Materials, Faculty of Chemistry, 83067 University of the Basque Country UPV/EHU, San Sebastián 20018, Spain; # KERBASQUE, Basque Foundation for Science, Bilbao 48009, Spain

**Keywords:** photocatalysis, carbon nitride, cobalt phthalocyanine, nanocomposite, hydrogen generation, photo-oxidation

## Abstract

The photocatalytic production of hydrogen stands out as a promising strategy to convert and store solar energy as chemical energy in the form of a sustainable energy carrier. In the present study, a hybrid photocatalyst based on cobalt phthalocyanine (CoPc) coupled with polymeric carbon nitride (CN) is synthesized using a simple, cost-effective, and upscalable method. Both components are held together in the hybrid nanocomposite via π–π interactions, as shown by detailed structural and optical characterization. The synergistic interaction between both components, CN, a metal-free semiconductor, valued for its stability and tunable electronic properties, and CoPc, known for its excellent light absorption and electronic properties, is evidenced in a proof-of-concept photocatalytic reaction: the photo-oxidation of benzyl alcohol (BzOH) to benzaldehyde (BzO). Chemical trapping reagents were employed to elucidate the reaction mechanism, showing favorable recombination dynamics of the hybrid photocatalyst (CoPc/CN) compared to the individual components. Furthermore, photocatalytic hydrogen production was conducted in an aqueous solution using triethanolamine (TEOA) as an electron donor, with the optimized CoPc/CN nanocomposite producing 1136.5 μmol h^–1^ g_cat_^–1^ of H_2_, achieving a 50% higher hydrogen yield compared to pristine CN. These results contribute to the design of high-performance photocatalytic materials for promising solar-to-X transformations.

## Graphical abstract

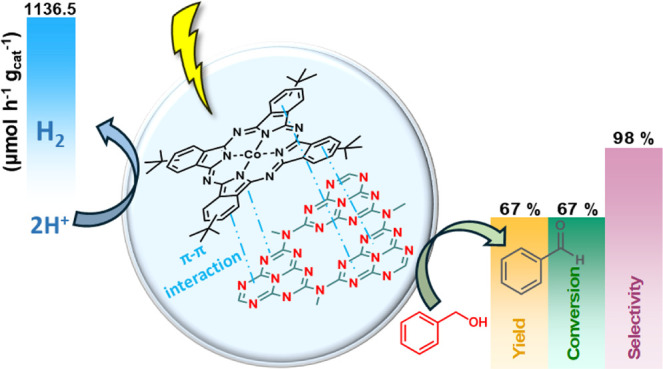

## Introduction

Hydrogen is considered a valuable feedstock for chemical synthesis and a clean, versatile energy carrier for transportation, power generation, and energy storage. The International Energy Agency (IEA) predicts that hydrogen demand will increase by 4 to 10 times and could potentially cover 10–25% of the world’s energy needs by 2050.^1^ In this context, sustainable processes for large-scale H_2_ generation are urgently needed, and photocatalytic hydrogen production with semiconductor nanoparticles is regarded as a promising solution, since only water and sunlight are used as feedstock and energy input, respectively, with zero emissions of greenhouse gases.^2–4^ Semiconductor materials like metal sulfides (CdS, CuS, and Znln_2_S_4_) and metal oxides (K_2_LaTa_2_O_6_N, TiO_2_, SrTiO_3_, MoO_3_, and CuO) have been widely applied as photocatalysts for H_2_ production.^5^ Yet, they suffer from photochemical corrosion, poor stability, photogenerated charge recombination, low visible light absorption, and high cost.^6^

Polymeric carbon nitride (CN) is a metal-free organic polymeric semiconductor, mainly composed of earth-abundant carbon and nitrogen. It is inexpensive, eco-friendly and exhibits good photochemical stability. CN has a bandgap of 2.7 eV and shows good photocatalytic activity under visible light irradiation.^7–10^ It is widely used as an organic photocatalyst for H_2_ generation,^11–13^ CO_2_ reduction,^14^ catalyzing organic reactions,^15^ and organic waste decomposition.^16,17^ As CN exhibits high charge recombination and suffers from limited visible light absorption and poor conductivity, it is typically heterostructured with other materials to overcome these limitations and to enhance its photocatalytic performance.^18^ These heterostructured photocatalysts have shown competitive performance for H_2_ evolution (CN_*x*_-(Mo_3_),^19^ NiS/CN,^20^ Au/CN,^21^ CN/Pt/TiO_2_,^22^ and Ni/CN^23^), photoreduction of CO_2_ ((PNS)-ZnO/CN,^24^ TiO_2_/CN,^25^ and CN/SnS_2_ ^26^), and decomposition of organic substrates like methylene blue, methyl orange, rhodamine B, aldehydes, and phenolic compounds (TiO_2_/CN,^27^ WO_3_/CN,^28^ and ZnO/CN^29^). Furthermore, when CN is combined with organometallic complexes like porphyrins,^30–32^ thioporphyrazines,^33^ and metal ions,^34^ enhanced photocatalytic activity is observed.

Particularly, the combination of pristine CN with phthalocyanines^35–42^ leads to enhanced photocatalytic activity. These inorganic compounds exhibit higher stability and activity due to high π-electron delocalization, allowing effective binding with CN. Metal phthalocyanines have been combined with CN, demonstrating enhanced photocatalytic activity (compared to pristine CN) when applied for CO_2_ reduction (CN/CoPc-COOH,^35^ CN/ZnPc,^36^ CoPc/B-CN,^42^ (CN/Co–N_4_ ^38^) H_2_ generation (Zn-tri-PcNc/CN,^39^ Zn-tri-PcNc/CN^40^) and organic waste degradation (CN/ZnO fibers,^41^ FePc/CN).^37^ Among the different metal-centered phthalocyanines previously studied, CoPc was selected for its ability to enhance the optical and catalytic properties of CN-based heterostructures, facilitating efficient electron transfer and significantly improving photocatalytic performance.

In the present study, we successfully combined tetra-*tert*-butyl cobalt (II) phthalocyanine (CoPc) with CN (Figure 1a) to form a novel hybrid nanocomposite photocatalyst, CoPc/CN. Both CN and CoPc present planar structures and bond together through π–π interactions.^43^ The nanocomposite was synthesized through mechanical grinding using a simple, cost-effective, and easily upscalable methodology. The suitable energy alignment and synergistic effect of CN and CoPc significantly enhance the photocatalytic performance by extending the light absorption range, improving charge separation efficiency, and increasing the density of active sites for photocatalytic reactions.^7,11^ The synthesized CoPc/CN hybrid photocatalyst was used in the selective photo-oxidation of benzyl alcohol (BzOH) to benzyl aldehyde (BzO) as a model photocatalytic reaction, since BzO is an important precursor for the synthesis of dyes, cosmetics, perfumes, polymers, and pharmaceuticals.^44^ Moreover, industrial BzO is prepared by the oxidation of toluene, which results in the formation of several byproducts, leading to low production yield and additional costly purification processes.^45^ The performed photocatalytic experiments showed a significant increase in the photo-oxidation of BzOH to BzO with a higher degradation rate. Furthermore, photocatalytic hydrogen production was conducted in an aqueous solution using triethanolamine (TEOA) as a sacrificial electron donor, and the optimized CoPc/CN nanocomposite catalyst demonstrated a hydrogen production rate of 1136.5 μmol h^–1^ g_cat_^–1^ (which is 1.5 times higher compared to pristine CN). This highlights the enhancement of the photocatalytic performance of the heterostructured CoPc/CN for H_2_ generation under visible light irradiation conditions.

**Figure 1:**
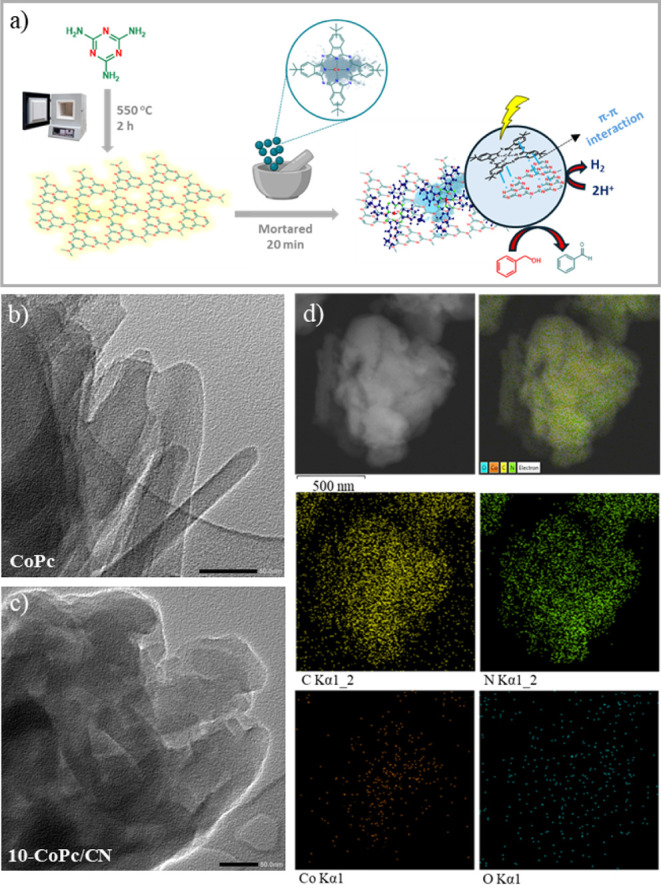
(a) Schematic illustration of the synthesis of CN and 10-CoPc/CN powders. TEM images of (b) CoPc and (c) 10-CoPc/CN. (d) TEM image and EDS elemental mapping for C, N, Co, and O. Scale bars in (b) and (c) are 50 nm.

## Results and Discussion

The CoPc/CN heterostructured photocatalyst was synthesized by following the procedure depicted in Figure 1a. CoPc was incorporated into the CN powder through pure mechanical grinding of different proportions of CoPc powders to obtain samples with different weight percentages (wt %), named 10-CoPc/CN (10 wt %) and 20-CoPc/CN (20 wt %) (see experimental details in Supporting Information). The incorporation of CoPc into CN resulted in a color change from yellow to teal green (Figure S1), suggesting enhanced visible light-harvesting efficiency. This modification extended the range of light absorption for the 10-CoPc/CN nanocomposite (Figure S2b) and consequently its photocatalytic potential. The synthesized heterostructured CoPc/CN is held together through weak π–π interactions, which contribute to the interfacial charge transfer upon light irradiation.^35^ The morphologies of both individual materials and the nanocomposite were investigated by scanning electron microscopy (SEM) and transmission electron microscopy (TEM), respectively. TEM images show that CoPc powder appears to be composed of elongated nanostructures approximately 30–40 nm thick, of nonuniform length, planar, and layered structures, characteristic of crystalline CoPc (Figures 1b and S3). On the other hand, CN showed the typical layered structure (Figure S4).^35,46^ Mechanical mixing of the CoPc/CN nanocomposite led to a coarser and less crystalline granular structure with higher roughness (Figure 1c). EDS elemental mapping showed the uniform dispersion of all the expected elements (carbon, nitrogen, cobalt, and oxygen) in the CoPc/CN heterostructure (Figure 1d), confirming the successful synthesis of the hybrid photocatalyst. SEM images of pure CN (Figure S5) evidence the layered structure of this material, while these CN layers appear embedded with CoPc in the CoPc/CN composite.^35,47^

The crystalline structure and phase purity of the different materials were assessed with X-ray diffraction (XRD) (Figure 2a). Pure CoPc exhibits a small peak at 2θ = 27 degrees, which corresponds to the (002) plane, due to π stacking between aromatic moieties,^48^ while the polymeric CN exhibits two distinct peaks at 2θ = 13.1 ° (100) and 27.6 ° (002) (JCPDS No. 87-1526), fully consistent with the hexagonal CN structure.^35^ The incorporation of CoPc into CN leads to a progressive decrease in the crystallinity of the hybrid nanocomposite, with π–π interaction occurring due to the effect of mechanical grinding.^9,49^

**Figure 2:**
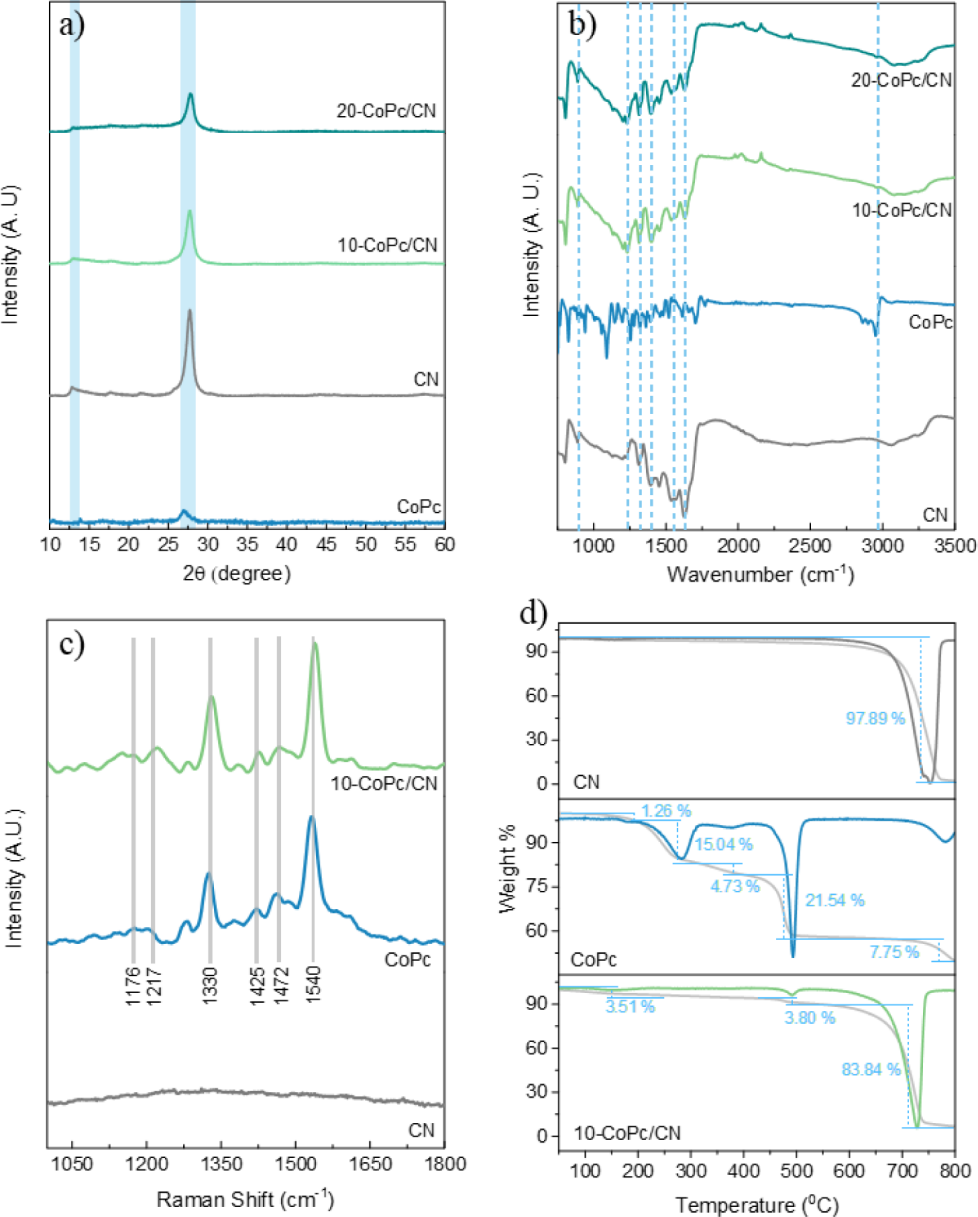
(a) XRD, (b) FT-IR, (c) Raman spectra, and (d) thermogravimetric analysis of CN, CoPc, 10-CoPc/CN, and 20-CoPc/CN powders.

Further structural information was obtained from Fourier transform infrared (FT-IR) spectroscopy (Figure 2b). The peaks at 888, 1227, 1313, 1397, 1539, and 1629 cm^–1^ are related to the characteristic stretching vibration modes of C–N and C=N of CN.^8,50^ On the other hand, the CoPc exhibits distinct peaks in the range of 1550–1650 cm^–1^, corresponding to the C=N and C=C bonds. The strong peak observed at 2954 cm^–1^ corresponds to the aromatic (C=C) stretching mode of *tert*-butyl phenyl group of CoPc.^9^ Due to the formation of π–π interactions between the CoPc and CN^49^ in the hybrid photocatalysts (10-CoPc/CN and 20-CoPc/CN), the intensity of the peaks related to CoPc and CN decreased.^51,52^

Raman spectroscopy (Figure 2c) of pure CN exhibits fluorescence, and hence, a broad spectrum is observed.^53^ In the case of CoPc, peaks at 1176 cm^–1^ and 1217 cm^–1^ correspond to C–H bending and vibrations in the phthalocyanine ring; the 1330 cm^–1^ peak corresponds to the in-plane stretching of the macrocyclic ring in CoPc, and the peaks around 1425–1540 cm^–1^ represent C =C and C=N stretching vibrations in the aromatic structure of the phthalocyanine ring.^54^ In the case of the hybrid photocatalyst (10-CoPc/CN), similar peaks corresponding to CoPc were observed, although with some variations in intensity and slight shifts. These changes arise from π–π interactions between CoPc and CN in the nanocomposite.^35,52,55^

The thermal stability of CN, CoPc, and CoPc/CN was studied by thermogravimetric analysis (TGA). A clear mass loss process was identified for CN between 670 and 770 °C (97.9%), related to the degradation of CN nanosheets at temperatures above 670 °C (Figure 2d). On the other hand, CoPc shows a multistep mass loss. At 30–150 °C, the 1.26% mass loss is most likely due to water desorption from the surface. As the temperature increases, three consecutive mass loss events appear from 200 to 300 °C (15.04%), 350 °C (4.73%), and 450–520 °C (21.54%), respectively. The 10-CoPc/CN photocatalyst features a water desorption process at low temperature (50–150 °C, 3.51%) and a loss process of 3.8% related to CoPc degradation, suggesting that integration with CN might slightly enhance its thermal stability. The weight loss continued until 770 °C due to the full degradation of the CN nanosheets. The overall degradation pattern aligns with the individual behavior of both CoPc and CN.^35^

The optical properties of CN, CoPc, and CN/CoPc were characterized by UV–vis spectroscopy (Figure S2a–c). The pristine CN material exhibits the π–π* and *n*–π* electronic transitions of the heptazine ring at 233–300 nm and 310–448 nm, respectively.^35^ The as-synthesized CoPc material shows two bands at 324 and 670 nm, corresponding to the B band (π–π*) and Q band (*n*–π*) of the phthalocyanine ring.^56^ The hybrid 10-CoPc/CN shows an enhancement of the π–π* absorption band of CN, along with the presence of the 550–720 nm band in the visible region, reflecting the successful incorporation of CoPc with CN. Moreover, the optical bandgaps (*E*_g_) of CN, CoPc, and CoPc/CN were determined by Tauc plots for indirect transitions (Figure S2a–c). The extrapolated *E*_g_ value for CN was 2.77 eV, while CoPc showed two bandgap transitions at 1.86 and 2.46 eV, respectively.^57^ On the other hand, two bandgaps of the 10-CoPc/CN hybrid photocatalyst were found at 2.05 and 2.72 eV, in good agreement with those observed for each individual compound, clearly indicating enhanced visible light absorbance.

Detailed information about the redox processes of CoPc was extracted from electrochemical measurements. In particular, the electrochemical properties of the monomer were determined using cyclic voltammetry (Figure S6). CoPc presents one reversible and one irreversible oxidation peak at 0.34 and 1.06 V vs Fc/Fc^+^, respectively. Moreover, a reversible reduction wave at −0.93 V vs Fc/Fc^+^ is detected. The reduction and oxidation behavior of cobalt phthalocyanines are related to the interaction between the phthalocyanine ring and the central metal.^57^ In the case of CoPc, the observed reduction peak corresponds to the reduction of the phthalocyanine ring, and the first and second oxidation peaks can be attributed to the oxidation of the central metal and the macrocycle, respectively.^58^ Additionally, to optimize the peak deconvolution in multielectronic electrochemical processes, differential pulse voltammetry (DPV) measurements were carried out. As observed, DPV peaks are in excellent agreement with those observed using cyclic voltammetry (Figure S6).

### Photo-Oxidation of Benzyl Alcohol (BzOH) to Benzaldehyde (BzO)

The photocatalytic performance of the synthesized CoPc/CN hybrid material was evaluated through the selective photo-oxidation of BzOH to BzO under ambient conditions as a proof-of-concept reaction (Figure 3a). When the reaction was carried out by irradiating with both UV and visible light (without using a UV-filter), the photocatalyst converted benzyl alcohol to benzaldehyde (55%) within 2 h. This has been reported and could be associated with the formation of singlet oxygen species in the solution generated by UV light.^50,59,60^ The same reaction was carried out in the presence of anthracene, which acts as a singlet oxygen quencher, and it led to a decrease in the yield of BzO (10%), confirming the generation of singlet oxygen as a reactive intermediate under UV irradiation. Then, the photocatalytic reactions were performed under visible light irradiation (using a UV-filter), and the calculated yield, conversion, and selectivity percentages for the targeted reaction using CN, CoPc, 10-CoPc/CN, and 20-CoPc/CN are presented in Figure 3b. Among these, the 10-CoPc/CN nanocomposite exhibited the best photocatalytic performance, achieving 67% yield, 67% conversion, and 98% selectivity. Doubling the concentration of the photocatalyst (d-10-CoPc/CN) had no significant effect on performance (Figure S7). However, the 20-CoPc/CN nanocomposite showed a drop in BzO yield to 54% (Figure 3a). In comparison, CoPc and CN alone produced lower yields of 28% and 38%, respectively, with reduced conversion and selectivity relative to the hybrid nanocomposite. Therefore, 10-CoPc/CN demonstrated superior photocatalytic activity in the photo-oxidation of BzOH to BzO and was selected for further experiments involving scavenging reagents and photodegradation of BzOH, monitored through UV–vis spectroscopy.

**Figure 3:**
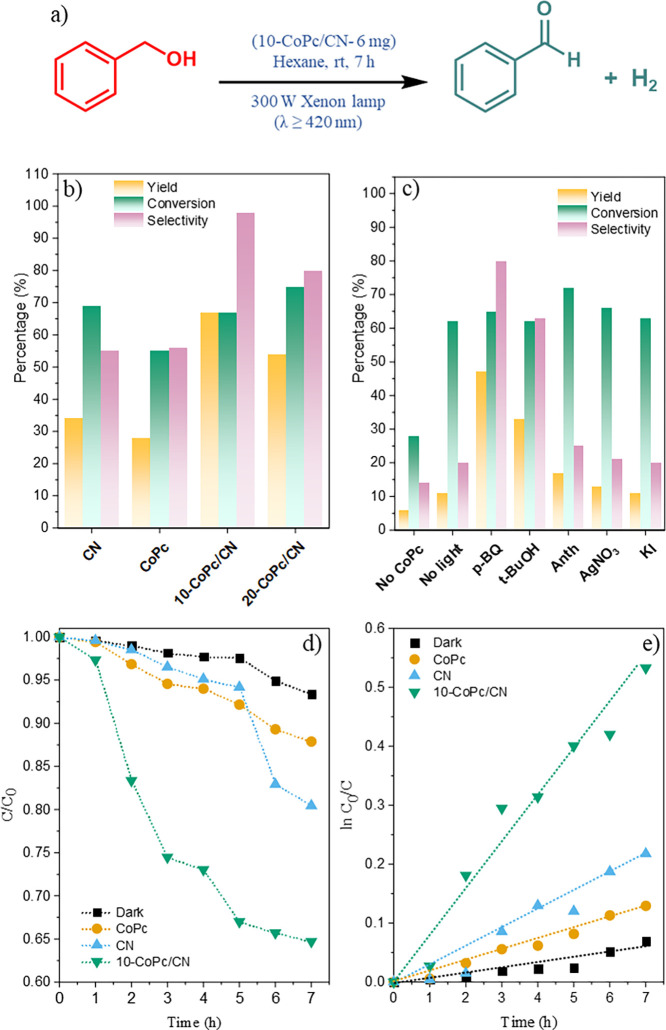
(a) Reaction conditions for the photocatalytic oxidation of BzOH to BzO. Yield, conversion, and selectivity for (b) photocatalytic oxidation of BzOH to BzO and (c) photocatalytic experiments with different scavenging reagents, photocatalytic degradation curves for BzOH degradation for the different studied materials (d) *C*/*C*_0_ vs time and (e) ln *C*/*C*_0_ vs time. All of the reactions were carried out in hexane at room temperature and by irradiating with visible light (xenon lamp, λ ≥ 420 nm).

The photocatalytic experiments under N_2_ bubbling showed no BzO formation from BzOH oxidation, confirming that O_2_ is essential for the generation of active singlet oxygen species. Additionally, after removing the photocatalyst at 4 h and continuing the reaction for 3 more hours, we observed a very low yield, indicating that BzOH autophotooxidation does not occur.

The photocatalytic experiments were carried out using 10-CoPc/CN and by adding different scavenging reagents like potassium iodide (KI), silver nitrate (AgNO_3_), 1,4-benzoquinone (p-BQ), anthracene (Anth), and *tert*-butanol (*t*-BuOH) (Figure 3c) in order to understand the mechanistic pathway of the reaction.^43,44^ In the absence of a photocatalyst, a trace amount of BzO is formed. When the reaction is carried out in the absence of light, there is a small increase in the yield (∼10%) with 60% conversion and 20% selectivity, which may be associated with the binding of BzOH with the photocatalyst, but the reaction cannot proceed further due to the absence of light. When p-BQ is added as a scavenging reagent, the conversion of BzOH to BzO is 65% with 45% yield and higher selectivity, due to the elimination of the superoxide anion radical (O_2_^•–^) as reactive intermediate.^63^ In the case of *t*-BuOH, which acts as a hydroxyl radical scavenger (OH^•^), there is a decrease in the formation of BzO (35% yield) with a conversion of 60%, which can be related to the competition between *t*-BuOH and benzyl alcohol in binding with the photocatalyst.^61^ Also, since the reaction is carried out in an aqueous-free environment, the formation of OH^•^ as a reactive intermediate can be eliminated.^43^ In the case of anthracene as a quenching reagent, there is a larger decrease in the formation of benzaldehyde (11%), which clearly shows that during the reaction, singlet oxygen (^1^O_2_) is generated, which gets trapped by the anthracene and slows down the formation of BzO.^62^ Further, the formation of BzO was very low when KI (11%) and AgNO_3_ (13%) were added and used as hole and electron trap reagents, respectively. In all of the above cases, benzoic acid is not observed, suggesting that the overoxidation of the formed products does not occur.^61^

Consequently, it is believed that CoPc acts as a photosensitizer, harvesting the incident light (550–700 nm) and promoting the charge transfer to CN.^38^ The surface of CN favors H-bonding with the OH group of benzyl alcohol, which facilitates the adsorption of BzOH on the surface of the photocatalyst, followed by a reaction with the photogenerated holes and ^1^O_2_ (the holes from CN are transferred to CoPc and generate ^1^O_2_), leading to the corresponding photocatalytic oxidation of BzOH to BzO.^61,64,65^ Hence, the holes and ^1^O_2_ generated by the photocatalyst catalyze the selective photo-oxidation of BzOH to BzO.

The degradation rate of BzOH was monitored through the characteristic absorption band (Figure S8a–d), and the change in the absorbance of BzOH (shown as *C*/*C*_0_) was measured for 7 h. The results are shown in Figure 3c, where *C*_0_ is the initial concentration and *C* is the concentration at the defined time intervals. The degradation rate of BzOH was low for all the reactions until 4 h, which could be associated with the binding of BzOH with the photocatalyst, initially consuming the photogenerated holes and then showing a rapid increase in product formation until 7 h. The 10-CoPc/CN nanocomposite shows a pseudo-first-order rate constant (*k*) of 1.27 × 10^–3^ min^–1^ which is about 2.5 and 4 times higher than that of CN (0.516 × 10^–3^ min^–1^) and CoPc (0.307 × 10^–3^ min^–1^), respectively.^66^

To evaluate the stability of the photocatalyst, XRD and FT-IR measurements were carried out for the best-performing material (10-CoPc/CN) before and after the photocatalytic experiments. The results are shown in Figure S9a,b. Minimal changes were identified, with only a slight decrease in intensity, which could be attributed to the leaching of a small amount of Co species from the photocatalyst.

The photocatalyst was recovered and further used for four consecutive experiments (Figure S10). The 10-CoPc/CN photocatalyst exhibited yields of 61%, 54%, 41%, and 34%, respectively, indicating a 50% loss of yield over the successive four runs. The chemical evolution of the surface of 10-CoPc/CN samples due to catalytic activity was further investigated by using X-ray photoelectron spectroscopy (XPS). Figure 4a presents the Co 2p spectra of the catalyst both before (blue) and after (red) four cycles of photocatalytic oxidation of BzOH to BzO. The spectra exhibit a spin–orbit split doublet with peaks at 796.8 eV (Co 2p_1/2_) and 781.2 eV (Co 2p_3/2_). Both components provide equivalent chemical information, with a binding energy difference (ΔE) of 15.6 eV between them. This value matches that measured for pure CoPc (reference spectra in light blue) and is consistent with previously reported literature values.^67^ The preservation of the Co 2p binding energy positions relative to pure CoPc, even after mixing with CN, suggests the absence of binding interactions between the Co species and the CN matrix. After the reaction, the Co 2p_1/2_ and Co 2p_3/2_ components in the postreacted 10-CoPc/CN sample retain the same binding energies as those in the prereaction material, which suggests that the environment around Co remains unchanged. A notable decrease in the Co 2p intensity is observed, accompanied by a decrease in the intensity of the N 1s signal (Figure 4b) after the reaction. To investigate compositional changes before and after the cycling photocatalytic process, we analyzed the ratio N_CoPc_/N_CN_ in the 10-CoPc/CN compound, where N_CoPc_ and N_CN_ represent the partial N 1s area contributions to the total N 1s area from the CoPc molecules and CN matrix, respectively. The N_CoPc_/N_CN_ ratio decreases from 0.23 ± 0.08 before the reaction to 0.05 ± 0.04 afterward, indicating a 4-fold reduction in the relative amount of CoPc within the CN matrix. Given the catalytic activity of CN alone, the observed 50% yield loss in the CoPc/CN compound can reasonably be attributed to CoPc detachment during the reaction.

**Figure 4:**
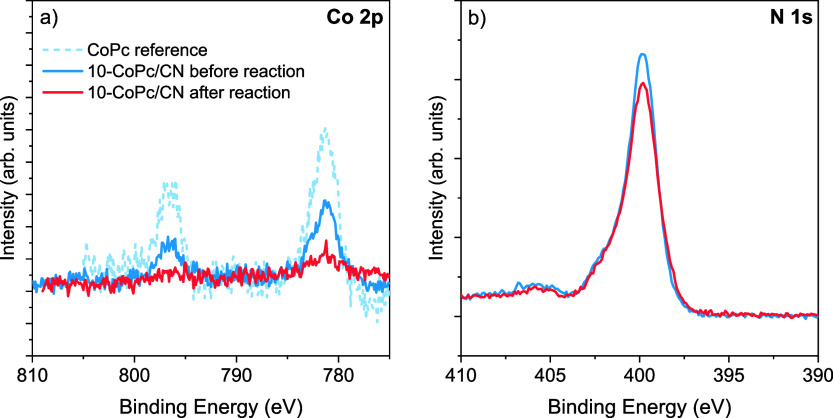
HR XPS spectra of (a) Co 2p and (b) N 1s regions of 10-CoPc/CN measured before (blue) and after (red) four cycles of photocatalytic oxidation of BzOH to BzO. In (a), the reference Co 2p spectrum of CoPc has been added (light blue, dashed line).

### Evaluation of the Photocatalyst Performance for H_2_ Production

The performance of the nanocomposites for the photocatalytic production of H_2_ was carried out in an aqueous solution containing chloroplatinic acid (H_2_PtCl_6_) and triethanolamine (TEOA) as a sacrificial oxidation reagent. The H_2_ evolution over time using the heterostructured photocatalysts is shown in Figure 5a. The photocatalytic H_2_ generation performance demonstrated a marked enhancement with the hybrid materials compared to CN, as CN achieved a rate of 774.5 μmol h^–1^ g_cat_^–1^, whereas the hybrid materials 10-CoPc/CN and 20-CoPc/CN exhibited significantly higher rates of 1136.5 μmol h^–1^ g_cat_^–1^ and 1003 μmol h^–1^ g_cat_^–1^, respectively (Figure 5b). This means that the H_2_ produced when 10-CoPc/CN is evaluated is 32% and 13% higher compared to CN and 20-CoPc/CN, respectively. The hybrid photocatalyst can effectively increase the charge transfer kinetics from the photocatalyst to the solution, leading to a larger production of H_2_, compared to pristine CN.^2,3,43^ The optimal catalyst loading at 10% could be associated with the formation of a thin layer of CoPc on the surface of CN, which maximizes the photocatalytic activity by decreasing charge recombination. Further loading of CoPc (20-CoPc/CN) leads to the accumulation of CoPc, which may form a layer that is too thick on the CN surface, leading to decreased photocatalytic activity.^36,43^ The H_2_ generation using the same 10-CoPc/CN solution was carried out for 10 h in 5 runs (each run lasting 2 h), and H_2_ evolution was monitored every 20 min to check the stability and reproducibility of the hybrid material. The H_2_ generation remained almost constant for 5 cycles, which was around 1131.2 μmol h^–1^ g_cat_^–1^ during each cycle,^2^ demonstrating that the photocatalytic H_2_ generation using this hybrid CoPc/CN material could be upscaled for extended periods (Figure 5c). The apparent quantum yields of the nanocomposite CN/CoPc were found to be 0.46%, 0.57%, and 1.71% at 420, 550, and 700 nm, respectively. This shows the advantage of the CN/CoPc photocatalyst as a potential candidate for hydrogen evolution from water under visible light irradiation.^36,68^ In addition, comparable performance for hydrogen production has been reported in the literature for the best-performing catalysts based on phthalocyanine hybrid materials, as shown in Table 1.

**Figure 5:**
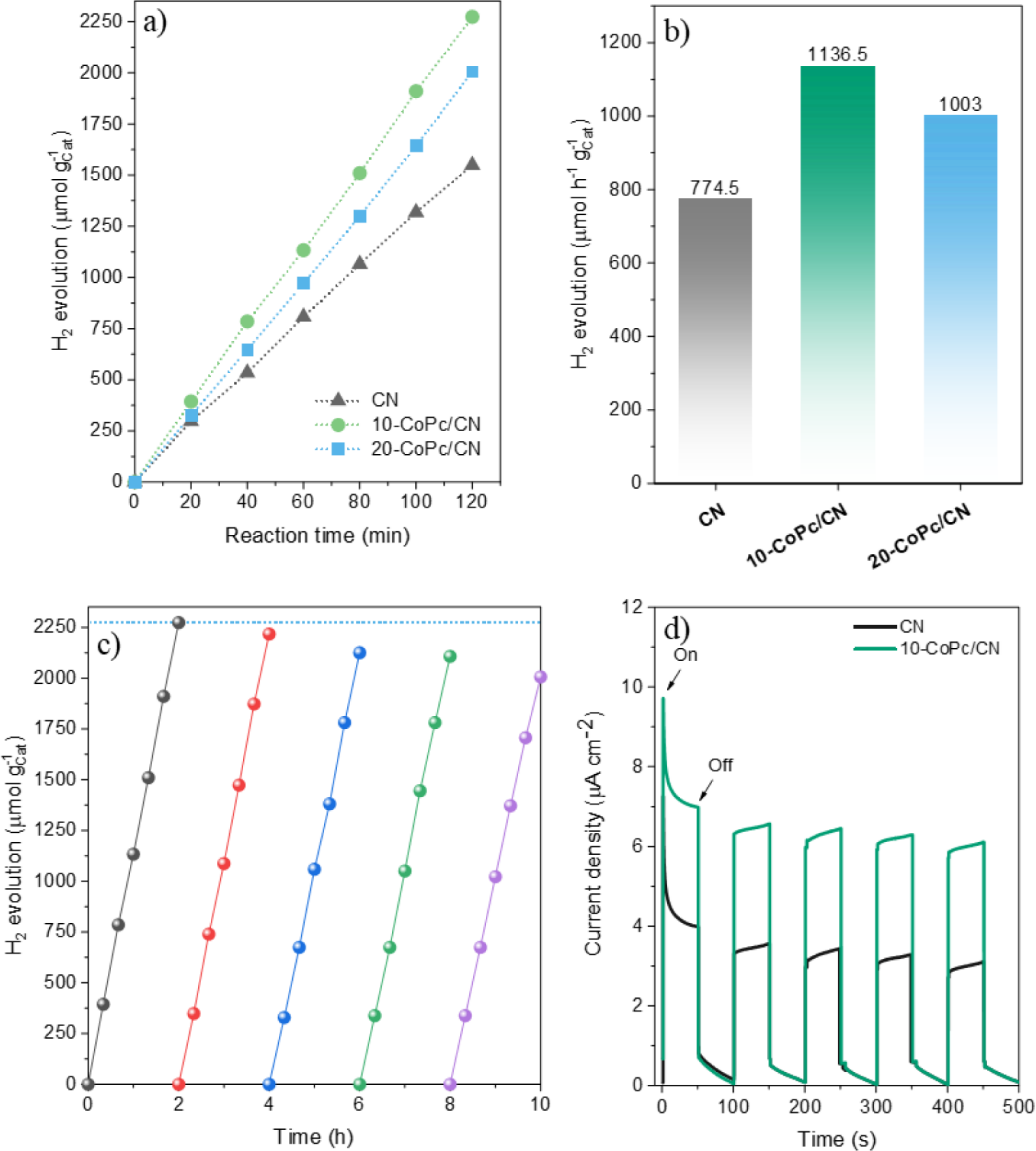
(a) H_2_ evolution over time and (b) rate of H_2_ evolution using CN, 10-CoPc/CN, and 20-CoPc/CN. (c) H_2_ evolution using 10-CoPc/CN for 10 h under 1 sun illumination in an aqueous solution 1with TEOA. (d) Chronoamperometry of CN and 10-CoPc/CN under 1 sun in an aqueous solution of 0.1 M Na_2_SO_3_.

**Table 1:** Comparison of Photocatalytic H_2_ Production for Metal Phthalocyanines

Catalyst	Sacrificial electron donor	Light source	Irradiation time (h)	H_2_ Production (μmol h^–1^ g_cat_^–1^)	Reference
N-usRGO/SiPc/Pt	TEOA	UV 254 nm halogen	6	240.6	^69^
Zn-tri-PcNc/CN	Ascorbic Acid	300 W xenon arc lamp (λ ≥ 500 nm)	3	125.2	^36^
Zn-PCH@TiO_2_	TEOA	300 W xenon arc lamp (λ ≥ 450 nm)	5	150.66	^11^
LI-4/CN/Zn-tri-PcNc	TEOA	300 W xenon arc lamp (λ ≥ 420 nm)	4	24.6	^41^
Zn-tri-PcNc-2-Pt/CN	Ascorbic Acid	300 W xenon arc lamp (λ ≥ 500 nm)	10	97.66	^70^
Zn-tri-PcNc-2-Pt/g-CN	Ascorbic Acid	300 W xenon arc lamp (λ ≥ 500 nm)	10	132	^71^
MgPc/Pt/mpg-CN	TEOA	300 W xenon lamp	10	500	^43^
CoPc/TiO_2_/Pt	TEOA	300 W xenon arc lamp (λ ≥ 420 nm)	8	3328	^2^
ZnPc/CN	TEOA	300 W xenon arc lamp (λ ≥ 420 nm)	5	14	^3^
NiPc@GO/TiO_2_	TEOA	300 W xenon arc lamp (λ ≥ 420 nm)	3	2760	^72^
CuPc/ZnIn_2_S_4_	TEOA	300 W xenon lamp	4	151.2	^73^
Mn_0.5_Cd_0.5_S@CuPc	Na_2_SO_3_	300 W xenon lamp	4	2140	^74^
10-CoPc/CN	TEOA	300 W xenon lamp (λ ≥ 420 nm)	2	1136.5	**This study**

A plausible mechanistic pathway is proposed in Scheme 1: Initially, light absorption at the CoPc moiety promotes electron excitation from the HOMO to the LUMO level of CoPc, which acts as a photosensitizer. These photoexcited electrons can be transferred from the CoPc to the CN photocatalyst, since the conduction band level of CN (−1.10 V vs NHE) is more positive compared to that of CoPc (−1.43 V vs NHE). The chloroplatinic acid (H_2_PtCl_6_) which acts as a cocatalyst is in contact with the surface of the photocatalyst and can be reduced to metallic Pt by the photoexcited electrons, which in turn reduces water into H_2_. TEOA is used as a sacrificial electron donor, which injects electrons into the CoPc during the photocatalytic reaction.^2,3,11^

**Scheme 1:**
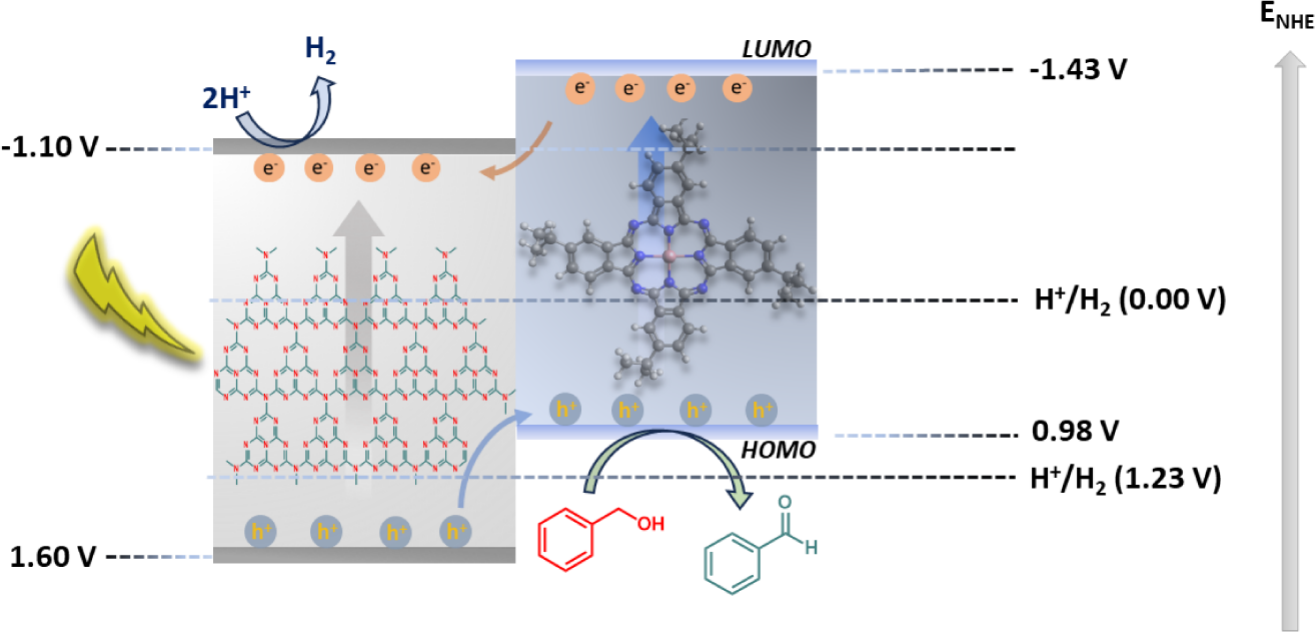
Energy Band Diagram of CoPc/CN, Illustrating the Proposed Mechanism for the Photocatalytic Oxidation of BzOH to BzO and for H_2_ Production

Finally, photoelectrochemical (PEC) experiments were carried out. The observed photocurrent densities provide a direct indication of the photoanode efficiency to drive the oxidation of TEOA, with a subsequent hydrogen evolution reaction (HER) at the cathode. As shown in Figure 5d, the CN electrode generated a photocurrent density of 4 μA cm^–2^, whereas the 10-CoPc/CN electrode exhibited a significantly higher photocurrent density of 7 μA cm^–2^, representing an approximate 1.75-fold improvement. This enhanced performance is attributed to the higher oxidation potential of CoPc/CN, due to its extended visible light absorption, more favorable recombination dynamics, and consequently improved charge transfer properties.^2,69^

## Conclusions

A novel CoPc/CN nanocomposite photocatalyst was successfully prepared by a simple, cost-effective, and scalable synthesis, where the incorporation of CoPc significantly enhanced the photocatalytic performance of pristine CN. This improvement is attributed to the increased light-harvesting efficiency of the hybrid photocatalyst and more efficient charge separation. The photocatalytic activity of the CoPc/CN nanocomposite was evaluated by oxidizing benzyl alcohol to benzaldehyde and generating hydrogen under visible light irradiation. The results demonstrated a substantial enhancement in both benzyl alcohol degradation and hydrogen production yield (1.8 and 1.5 times higher compared to the pristine CN material, respectively), highlighting the synergistic effect of the nanocomposite photocatalyst. This enhanced performance is mainly due to the more efficient electron transfer between CN and CoPc, which leads to more favorable recombination dynamics in the nanocomposite. This study not only demonstrates the potential of the CoPc/CN nanocomposite for environmental remediation and sustainable energy applications but also provides design guidelines for the development of alternative high-performance photocatalytic materials.

## Supplementary Material



## Notes

Experimental details, photograph of the powders, UV–vis characterization, TEM-EDS, mass spectrum, electrochemical characterization of CoPc, photocatalytic oxidation of BzOH to BzO, tracking of benzyl alcohol photooxidation, recycling test of the nanocomposite (PDF)
