# Supplementary material for: Hybrid Carbon Nitride/Cobalt Phthalocyanine Nanocomposites for Efficient Photocatalytic Hydrogen Generation
Source: ACS Appl Energy Mater. 2025 Apr 4;8(8):5056–66. doi: 10.1021/acsaem.4c03257 (PMC13295088; doi:10.1021/acsaem.4c03257)
Supplement: Supplementary file 1 [file ae4c03257_si_001.pdf]

Supporting Information

Hybrid carbon nitride/cobalt phthalocyanine nanocomposites for efficient photocatalytic hydrogen generation.

Lakshman Sundar Arumugam,[1] Javier E. Durantini,[1] Jorge Follana-Berná,[2] Frederik Schiller,[3,4] Ane Etxebarria,[5] Lorenzo Forzanini,[1] Sara Barja,[3,4,5,6,] Ángela Sastre-Santos,[2,*] Sixto Giménez,[1,*]*

[1] Institute of Advanced Materials, Universitat Jaume I, Avinguda de Vicent Sos Baynat, s/n, 12006 Castelló de la Plana, Spain

[2] Área de Química Orgánica, Instituto de Bioingeniería, Universidad Miguel Hernández de Elche, Elche 03202, Spain

[3] Centro de Física de Materiales CFM (UPV/EHU-CSIC), University of the Basque Country UPV/EHU, 20018 San Sebastián (Spain)

[4] Donostia International Physics Center DIPC, 20018 San Sebastián, Spain

[5] Department of Polymers and Advanced Materials, Faculty of Chemistry, University of the Basque Country UPV/EHU, 20018 San Sebastián (Spain)

[6] KERBASQUE, Basque Foundation for Science, 48009 Bilbao, Spain

* Corresponding authors: sara.barja@ehu.eus, asastre@umh.es, sjulia@uji.es

**EXPERIMETAL DETAILS**	**S-3**
**S1. PHOTOGRAPH OF THE CoPc, CN and CoPc/CN NANOCOMPOSITE POWDERS**	**S-9**
**S2. UV-VIS CHARACTERIZATION**	**S-10**
**S3. TEM-EDS AND MASS SPECTRUM FOR CoPc**	**S-11**
**S4. TEM IMAGE AND EDS SPECTRUM FOR CN**	**S-12**
**S5. SEM IMAGES FOR CoPc, CN and CoPc/CN**	**S-13**
**S6. ELECTROCHEMICAL CHARACTERIZATION**	**S-14**
**S7. PHOTOCATALYTIC OXIDATION OF BzOH TO BzO.**	**S-15**
**S8. TRACKING OF BENZYL ALCOHOL PHOTOOXIDATION THROUGH UV-Vis ABSORPTION SPECTROSCOPY**	**S-16**
**S9. XRD AND FT-IR MEASUREMENTS OF CoPc/CN BEFOR AND AFTER THE PHOTOCATALYSIS EXPERIMENT.**	**S-17**
**S10. RECYCLING TEST OF THE PHOTOCATALYTIC NANOCOMPOSITE**	**S-18**

EXPERIMENTAL DETAILS

Chemical and Materials.

Melamine (99%), Benzyl alcohol (99%), Hexane (99%) Triethanol amine (99.5%), Methanol (99.9%), Ethanol (99%), Nafion 117 were purchased from Sigma Aldrich and were used without any further purification.

Synthesis of graphitic Carbon Nitride powder

Polymeric carbon nitride (CN) powder was prepared according to the previously reported literature procedure, with a few modifications.[1] Melamine (2 g) was placed in a ceramic crucible, which was completely covered, and then placed in a muffle furnace. It was heated in air at 20 ºC min^{-1} until 550 ºC and maintained at that temperature for 2 hours. Afterwards, the crucible was cooled to room temperature, the yellowish CN powder was grinded using a mortar and pestle, and then dispersed in methanol and centrifuged three times (each 10 minutes, 1000 rpm). The resulting CN powder was then characterized and used as it is.

Synthesis of CoPc monomer

200 mg (1.1 mmol) of 4-*tert*-butylphthalonitrile, 35 mg (0.3 mmol) of $CoCl_2$ and 2 drops of DBN were dissolved in 5 mL of DMAE and heated at 160°C overnight. The mixture of different regioisomers was cooled to room temperature, washed with MeOH and purified by column chromatography (Hexane/Dioxane 3:1), yielding 90 mg (42%) of CoPc (mixture of isomers). HR-MALDI-TOF (dithranol) m/z $[M]^+$ for $C_{48}H_{48}CoN_8$: calcd, 795.3328; found, 795.3308. UV-Vis ($CHCl_3$) λ_{max}/nm (log ε): 328 (4.86), 605 (4.52), 670 (5.24) ν_{max} (KBr)/cm^{-1}: 2954, 1616, 1525, 1484, 1408, 1363, 1326, 1281, 1258, 1202, 1156, 1094, 1056, 942, 891, 826. Due to the paramagnetic character of cobalt, there are no signals in ^1H NMR for the CoPc core.

Synthesis of CoPc/CN composite powders.

The CoPc/CN composite was prepared at different mass fractions of CoPc:CN. Samples containing 10 and 20 wt% of CoPc in CN (named 10-CoPc/CN and 20-CoPc/CN, respectively) were fabricated by mechanically grinding both compounds with a mortar and pestle for 20 minutes. The as-synthesized CoPc/CN powder was characterized and used without any further modification. Alternatively, we synthesized the heterostructured photocatalyst by mixing CN and CoPc in solution followed by

drying, but due to ineffective binding, the material had low stability and degraded quickly during the photocatalytic reaction.

Synthesis of CN, CoPc/CN electrodes.

The CN and CoPc/CN electrodes were prepared by ultrasonicating 1 mg of the sample in 1 mL of ethanol and 1 mL of Nafion® and a clear solution was obtained. Then, the solution was drop casted (60 μL) on top of Fluorine-doped tin oxide (FTO) glass and dried at 60 ^{0}C in an oven for 6 hours.[2]

Structural, Morphological, Optical, Electrochemical and Photoelectrochemical Characterization.

The morphology and composition of the powders were studied by high resolution transmission electron microscopy (HR-TEM) using a LaB6 JEOL JEM 2100 plus equipped with Aztec TEM Ultim Max Oxford EDS analyser operating at 200 kV. Atomic resolution aberration corrected (AC) HAADF and iDPC STEM images and the corresponding EDS composition maps were obtained in a Spectra 300 Thermo Fisher microscope equipped with Super-X EDX detectors. The AC STEM was operated at 300 KeV. The crystalline structure of the samples was determined by X-ray diffraction (XRD) analysis using a Bruker-AXS X-ray diffractometer with a Cu Kα radiation (λ = 1.5418 Å) operating at a grazing incidence of 1 °, and at a scan rate of 3 ° min^{-1} for 2θ angles from 15 to 70 °.

Thermogravimetric analysis (TGA) measurements were performed on a NETZSCH TG 209F1 LIBRA instrument using a heating rate of 5 °C/min from room temperature to 800 °C, under air flow.

The morphology of the CN, CoPc and the CoPc/CN mixture were examined by field-emission scanning electron microscopy (FE-SEM) with a JSM-700F JEOL FEG-SEM system (Tokyo, Japan) equipped with an INCA 400 Oxford EDS analyzer (Oxford, UK) operating at 15 kV. Prior the FE-SEM measurements, the samples were sputtered with a 2 nm thick layer of Pt. Raman spectra were measured with a WITec Apyron confocal microscope using a 532 nm laser with a 1 mW power, a grating of 1800 g/mm, a BLZ = 500 nm and an optical objective Zeiss EC Epiplan-Neofluar Dic 50x/0.55. For FTIR spectroscopy, a Fourier FT/IR-6200 (Jasco) equipped with an *ATR Pro One* device and an IRT-3000 microscope was used. High-resolution mass spectrum was obtained

from a Bruker Microflex LRF20 matrix-assisted laser desorption/ionization time-of-flight (MALDI-TOF) using dithranol as matrix.

XPS characterization was carried out in a Specs GmbH system, which has a Phoibos 150 NAP analyzer, a μFOCUS 600 X-ray monochromator (spot size of 300 μm), and an Al Kα anode (1486.7 eV) as the X-ray source. The high-resolution spectra were collected at 20 eV pass energy. During measurements, the sample was at room temperature, and the system was under UHV conditions. The data was analyzed using the software CasaXPS (Casa Software Ltd, Teignmouth, UK).

UV-visible absorption spectra of the colloidal solution were recorded in absorbance mode with a Jasco V-780 Spectrophotometer. The optical response of the powders was recorded on a Lambda 1050+ spectrophotometer (Perkin Elmer). The bandgap of the different materials was obtained from Tauc plots (***Eq S1***), where *α, h, v*, E_g, and B are absorption coefficient, Planck constant, light frequency, optical bandgap, and the B is a constant, and m is 1/2 or 2 for indirect and direct allowed transition, respectively.[3] Since CN is a direct bandgap semiconductor m = 2 is used for the calculations in these samples.[4,5] From a plot $(\alpha hv)^2$ *versus* photon energy (*hv*): the intercept of the tangent to the x-axis gives a good approximation of E_g.

$$(\alpha h v)^m = B\left(hv - E_g\right) \qquad \textbf{\textit{Eq S1}}$$

Moreover, the reflectance spectra measured can be converted into the corresponding absorption spectra using the Kubelka–Munk function (F(R), equation), as proposed by P. Kubelka and F. Munk in 1931.[6]

$$F(R) = \frac{K}{S} = \frac{(1-R)^2}{2R} \qquad \textbf{\textit{Eq S2}}$$

Where $R = R_{sample}/R_{standar}$ is the reflectance of an infinitely thick specimen, while K and S are the absorption and scattering coefficients, respectively. Substituting F(R) for α in ***Eq S1*** results in the expression given in ***Eq S3***.

$$(F(R)\ hv)^m = B\left(hv - E_g\right) \qquad \textbf{\textit{Eq S3}}$$

Electrochemical studies (Cyclic voltammetry (CV), and differential pulsed voltammetry, DPV) were performed with a computer controlled Autolab potentiostat/galvanostat (PGSTAT302), using a three-electrode cell configuration, with a Pt working electrode, a large area Pt counter electrode, and a Ag wire pseudo-reference electrode. Concentrations of 0.5×10^{-3} M were used for the electrochemical

characterization of CoPc. CoPc was dissolved in dichloroethane (DCE) containing 0.10 M tetra-*n*-butylammonium hexafluorophosphate ($TBAPF_6$) as supporting electrolyte. Before each electrochemical test, the solution of CoPc was deoxygenated by argon bubbling. In all the electrochemical measurements, the Pt working electrode was mechanically cleaned between experiments by polishing with alumina paste (0.3 μm), followed by solvent rinses. After each voltammetric experiment, ferrocene was added as an internal standard.

The composites performance evaluation in the generation of H_2 through photo-catalyzed water splitting was carried out using the as synthesized CN and the different photocatalyst mixtures (10-CoPc/CN and 20-CoPc/CN) in 10 mL aqueous solution containing 50 μL of 8 wt% hexachloroplatinic acid solution (H_2PtCl_6) and 2 mL of triethanolamine (TEOA) as sacrificial reagent. The light source was a 300 W Xe lamp (Newport Oriel) with simulated AM 1.5 G solar irradiation, adjusting the light intensity to 100 $mW\cdot cm^{-2}$ using a Si photodiode sensor (Ophir photonics), along with UV cut-off filter ($\lambda \geq 420$ nm). The pH of the solution was found to be 7.2. H_2 evolution was quantified by gas chromatography (GC). The outlet gas was analyzed every 5 minutes by an Agilent Technologies AG-490 instrument (with thermal conductivity detector, μTCD together with a narrow-bore column).

Photoelectrochemical (PEC) experiments of the photoelectrodes were carried out at room temperature using a three-electrode electrochemical cell, using CoPc/CN photoelectrodes (1 cm^2) as working electrode, a Pt wire as counter electrode and Ag/AgCl as reference electrode. The cell was connected to a PGSTAT302 potentiostat/galvanostat to perform PEC measurements. The light source was a 300 W Xe lamp (Newport Oriel) with simulated AM 1.5 G solar irradiation, adjusting the light intensity to 100 $mW\cdot cm^{-2}$ using a Si photodiode sensor (Ophir photonics). During the tests, the photoanodes were illuminated from the electrode/electrolyte side (back illumination), with the electrode in contact with a solution of 0.1 M of Na_2SO_4 (pH= 7.2), the applied potential was measured against the Ag/AgCl reference electrode and converted into the reversible hydrogen electrode (RHE) by using the Nernst equation (***Eq S4***):

$$E_{RHE} = E_{\frac{Ag}{AgCl}} + E^{o}_{\frac{Ag}{AgCl}} + 0.0591V \times pH \qquad \textbf{\textit{Eq S4}}$$

$$E^{o}_{\frac{Ag}{AgCl}} = 0.1976V\ vs\ NHE\ at\ 25\ °C$$

The chronoamperometry experiments were performed by applying a potential of 0.5 V vs RHE for 500 s chopping the light every 50 seconds.

Photocatalytic oxidation of BzOH to BzO.

We targeted the selective oxidation of BzOH to BzO as a model photocatalytic reaction to validate the CoPc/CN photocatalyst, focusing on BzO as the most valuable oxidation product. For this reaction, we added 6 mg (10-CoPc/CN), (20-CoPc/CN), 12 mg of photocatalyst composite (d-10-CoPc/CN) and 1 mM BzOH in 6 mL of hexane with constant magnetic stirring (400 rpm) irradiated by a Xe lamp (300W) with UV cut-off filter ($\lambda \geq 420$ nm) for 7 hours and the aliquots were collected at 0, 1, 2, 3, 4, 5, 6, 7 hours. Additionally, control experiments using CN, without the photocatalyst and dark conditions were carried out. The collected samples were analyzed by gas chromatography (GC) (Shimadzu GC-2010) equipped with flame ionization detector. The capillary column (ZB-5MS) with 30 m length and 0.25 mm inner diameter with 0.5 μm film thickness was used for the analysis. The chromatograms were obtained by injecting 1 μL of sample with a split ratio of 8. The column temperature was set at 50 °C for 3 min and increased to 300 °C (rate 9 C/min) with a final holding time of 2 min.

In order to identify the main reactive oxygen species (ROS) responsible for BzOH conversion to BzO, various compounds were added as scavenging reagents. Potassium Iodide (KI) was used as h^+ scavenger, silver nitrate ($AgNO_3$) as e^- scavenger, 1,4-Benzoquinone (p-BQ) as $O^{2\bullet -}$ scavenger, anthracene (Anth) as $^1O_2$ and *tert*-butanol (t-B) as $^{\bullet}OH$ scavenger.

The degradation rate of BzOH was monitored through the characteristic absorption band **(Supporting Information, Figure S8a-d)** and the change in the absorbance of BzOH (showed as C/C_0) was measured for seven hours. The respective pseudo-first-order rate constants (k) of BzOH to BzO were obtained from the graph of $\ln(C_0/C)$ vs time, by using the equation $\ln(C_0/C) = kt$, where (t) is the reaction time.

Calculation of yield, selectivity and conversion % of BzO through photocatalytic oxidation of BzOH.

The yield, selectivity and conversion % of BzO obtained from gas chromatography were calculated using the following equations:

$$Conversion\ (\%) = \frac{\left[\frac{C_i - C_f}{C_i}\right]}{100} \qquad \textbf{Eq S5}$$

$$Yield\ (\%) = \frac{\left[\frac{C_0}{C_i}\right]}{100} \qquad \textbf{Eq S6}$$

$$Selectivity\ (\%) = \frac{\left[\frac{C_0}{C_0 - C_f}\right]}{100} \qquad \textbf{Eq S7}$$

Where C_i is the initial BzOH concentration, while C_f and C_o are the concentration of BzOH and BzO, respectively after 7 hours of photocatalytic reaction.

The apparent quantum efficiency (AQY) was measured using the same 300 W xenon lamps equipped with bandpass filters of 420, 550, and 700 nm, respectively. The AQY value was calculated by the following equation:

$$AQY(\%) = \frac{2\ x\ (number\ of\ evolved\ H\ molecules)}{number\ of\ incident\ photons}\ x\ 100$$

S1. IMAGES OF CoPc, CN AND CoPc/CN NANOCOMPOSITE POWDERS

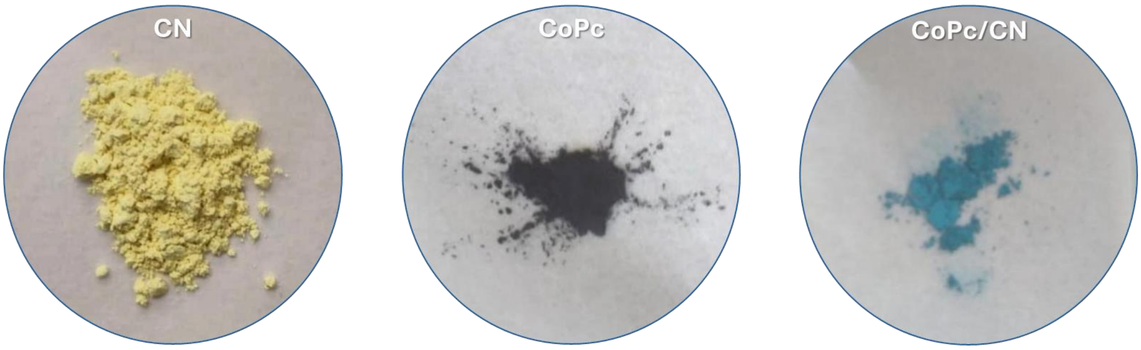

Figure S1. Photograph of CoPc, CN and CoPc/CN powders showing the incorporation of CoPc into CN resulted in a colour change from yellow to teal green.

S2. UV-VIS CHARACTERIZATION

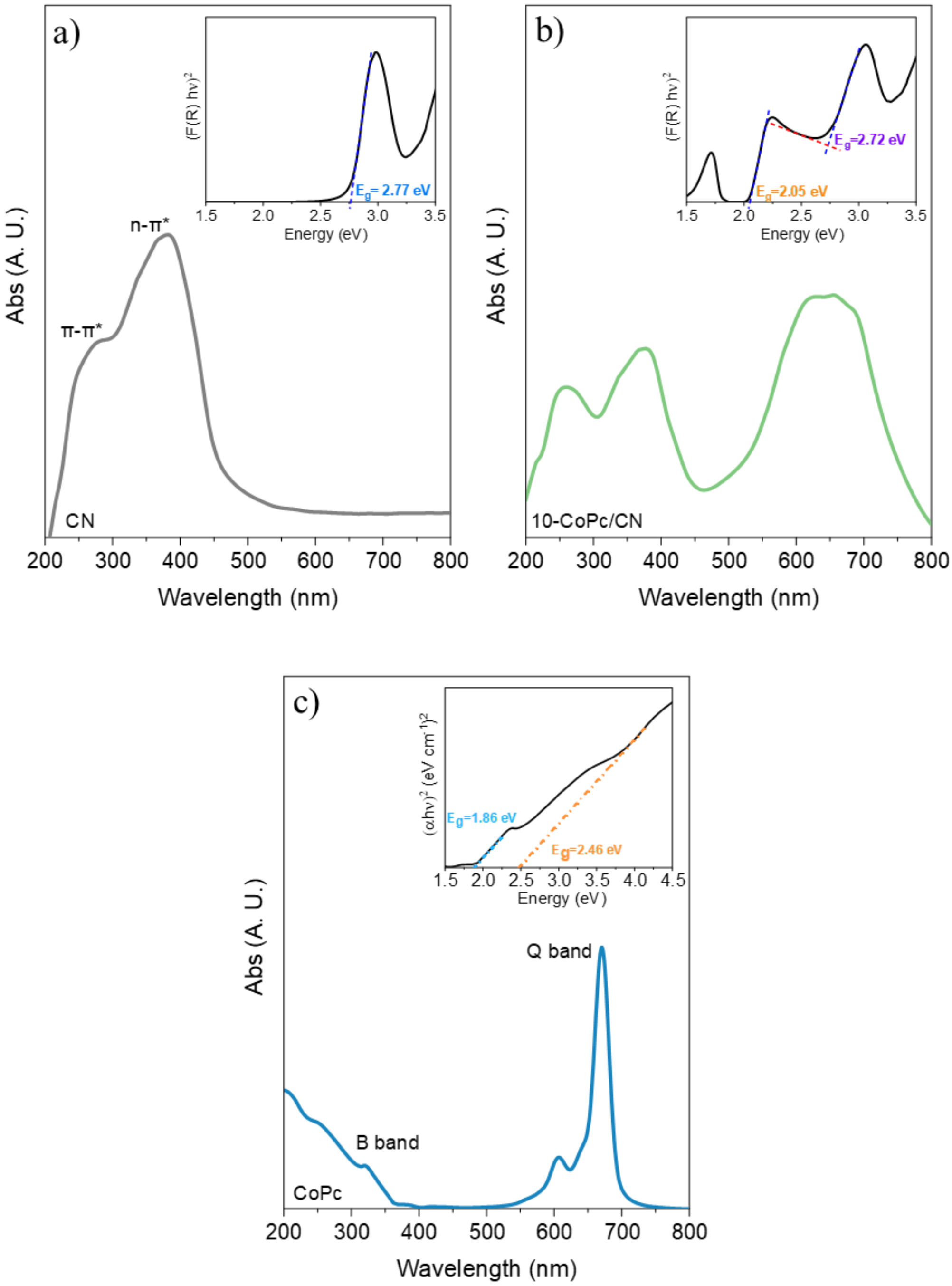

Figure S2. UV-Vis absorption spectrum and Tauc plots (insets) for nanocomposite powders of a) CN, b) 10-CoPc/CN and c) CoPc.

S3. TEM-EDS AND MASS SPECTRUM OF CoPc

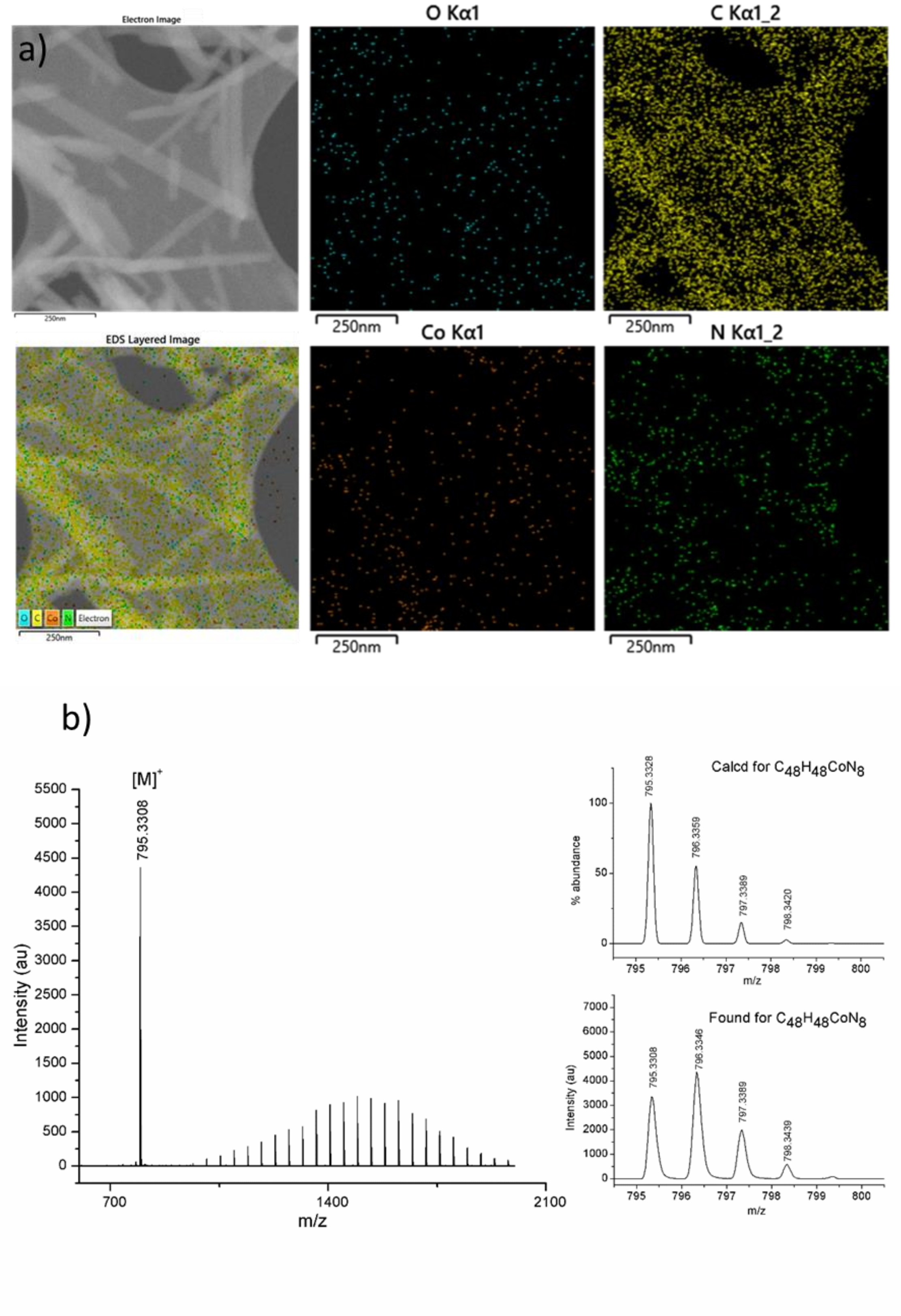

Figure S3. a) TEM elemental mapping patterns of CoPc and, EDS layered image, and the individual elemental mapping corresponding to C, N, Co and O and b) MALDI-ToF mass spectrum of CoPc.

S4. TEM IMAGES AND EDS SPECTRUM OF CN

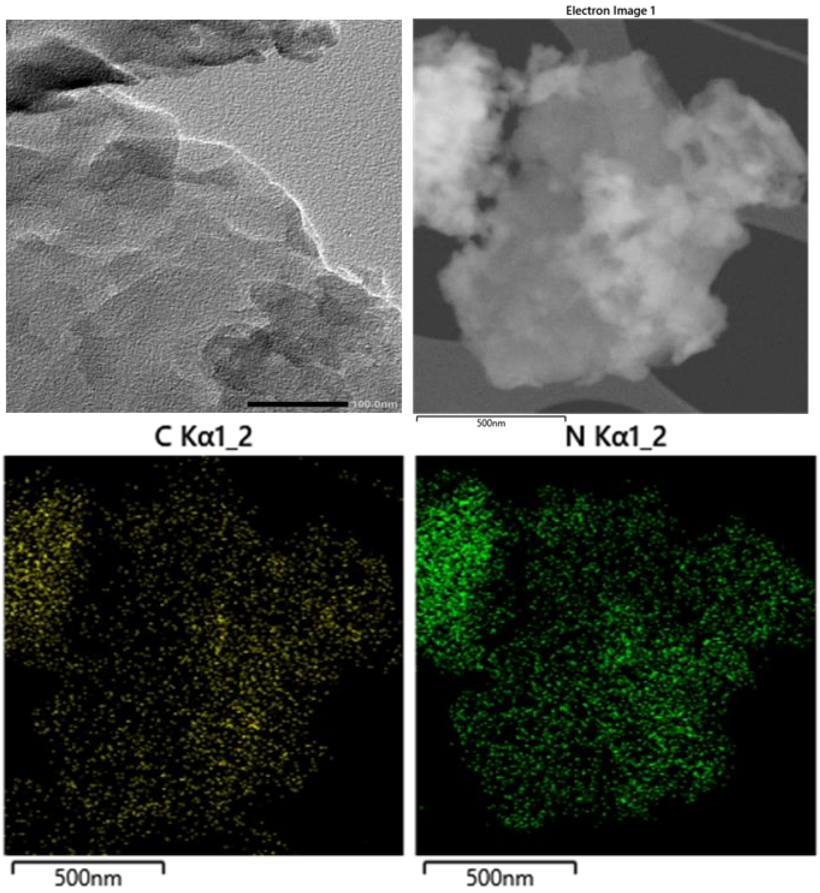

Figure S4. TEM images of CN, EDS elemental mapping patterns of CN and the individual elemental mapping corresponding to C and N.

S5. SEM IMAGES OF CN, CoPc, CoPc/CN

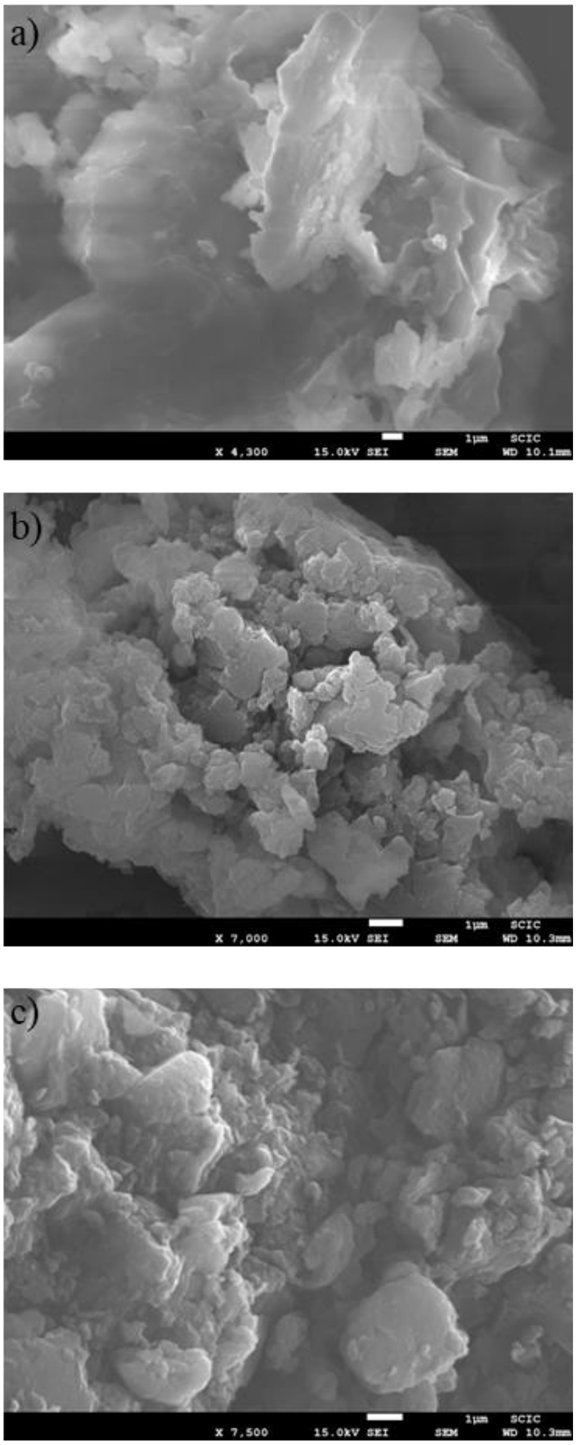

Figure S5. SEM images of a) CN, b) CoPc and c)10-CoPc/CN at different magnifications. Scale bar for all images is 1 μm.

S6. ELECTROCHEMICAL CHARACTERIZATION

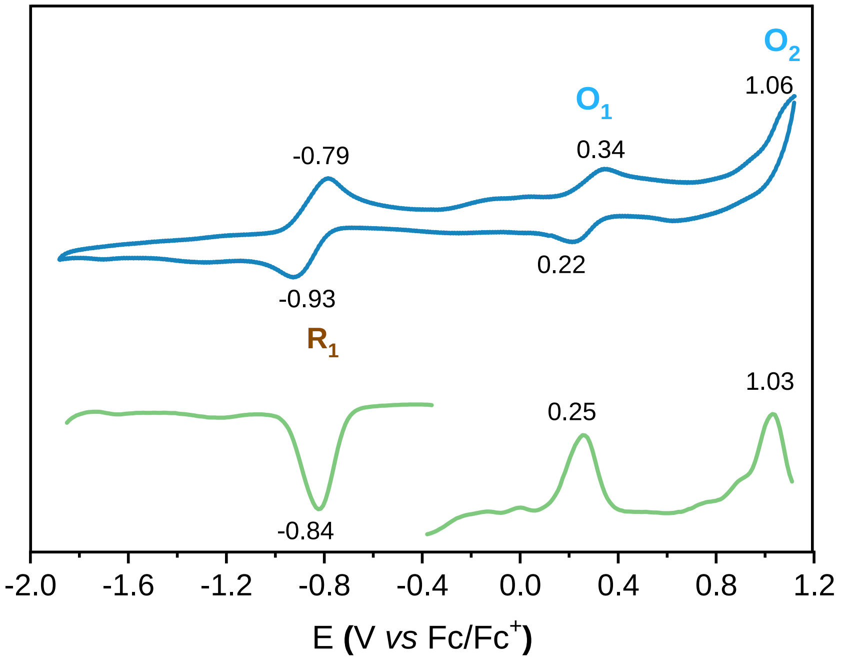

Figure S6. Cyclic Voltammogram (top) and Differential Pulse Voltammetry (bottom) of CoPc in 1,2-dichloroethane (DCE) containing 0.10 M tetrabutylammonium hexafluorophosphate ($TBAPF_6$). Pt working electrode, scan rate 100 mV/s.

S7. PHOTOCATALYTIC OXIDATION OF BzOH TO BzO.

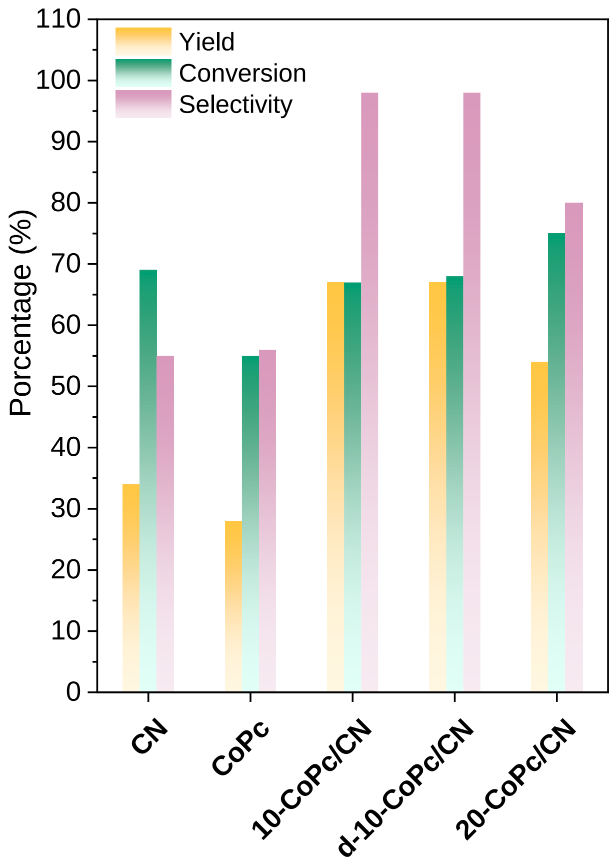

Figure S7. Yield, conversion and selectivity for the photocatalytic oxidation of BzOH to BzO of the different studied materials. Increasing the photocatalyst concentration by double (d-10-CoPc/CN) refers to 12 mg of photocatalyst composite (instead of 6 mg (10-CoPc/CN)) and 1 mM BzOH in 6 mL of hexane. The experiments were carried out with constant magnetic stirring (400 rpm) irradiated by a Xe lamp (300W) with UV cut-off filter ($\lambda \geq 420$ nm) for 7 h.

S8. TRACKING OF BENZYL ALCOHOL PHOTO-OXIDATION THROUGH UV-Vis ABSORPTION SPECTROSCOPY

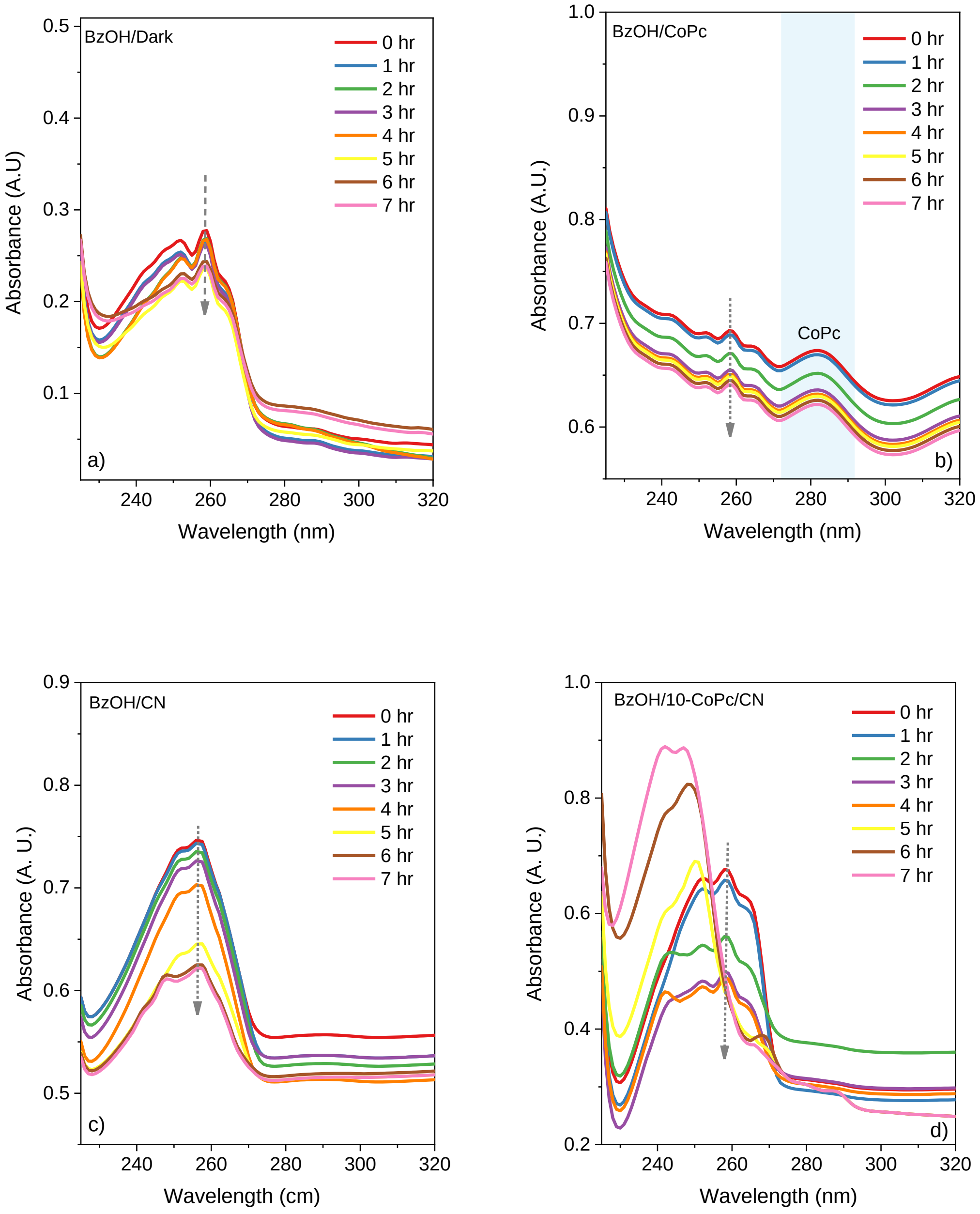

Figure S8. UV-Vis spectra at different times for the degradation of BzOH: a) In the dark, b) CoPc, c) CN and d) 10-CoPc/CN.

S9. XRD AND FT-IR MEASUREMENTS OF CoPc/CN BEFORE AND AFTER THE PHOTOCATALYSIS EXPERIMENT.

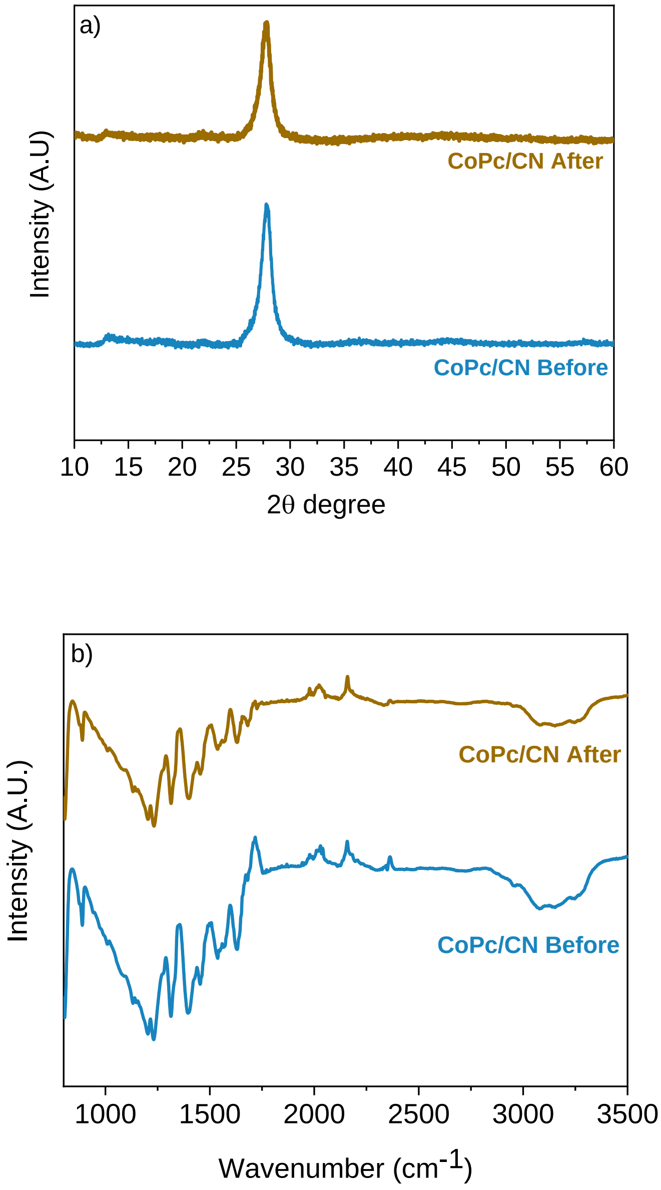

Figure S9. a) XRD and b) FT-IR spectra of the best performing material (10-CoPc/CN) before and after the photocatalysis experiment.

S10. RECYCLING TEST OF THE PHOTOCATALYTIC NANOCOMPOSITE

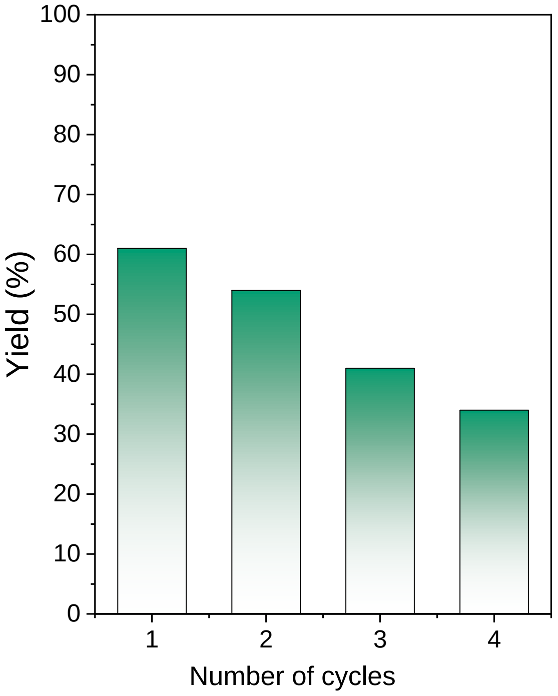

Figure S10. Recycling test of the 10-CoPc/CN photocatalytic nanocomposite after 4 cycles of photooxidation of BzOH to BZO.

References:

(1) Wang, X.; Maeda, K.; Thomas, A.; Takanabe, K.; Xin, G.; Carlsson, J. M.; Domen, K.; Antonietti, M. A Metal-Free Polymeric Photocatalyst for Hydrogen Production from Water under Visible Light. *Nat Mater* 2009, *8* (1), 76–80. https://doi.org/10.1038/nmat2317.

(2) Yu, H.; Xiao, P.; Wang, P.; Yu, J. Amorphous Molybdenum Sulfide as Highly Efficient Electron-Cocatalyst for Enhanced Photocatalytic H2 Evolution. *Appl Catal B* 2016, *193*, 217–225. https://doi.org/10.1016/j.apcatb.2016.04.028.

(3) Pankove, J. I.; Kiewit, D. A. Optical Processes in Semiconductors. *J Electrochem Soc* 1972, *119* (5), 156Ca. https://doi.org/10.1149/1.2404256.

(4) Gokul, V.; Swapna, M. N. S.; Ambadas, G.; Sankararaman, S. I. Thermal Lens Study of Hydrothermally Synthesised Graphitic Carbon Nitride Nanofluids for

Heat Transfer Applications. *Journal of Materials Science: Materials in Electronics* 2023, *34* (21). https://doi.org/10.1007/s10854-023-10991-w.

(5) Subin David, S. P.; Veeralakshmi, S.; Sakthi Priya, M.; Nehru, S.; Kalaiselvam, S. Room-Temperature Chemiresistive g-C3N4/Ag2ZrO3 Nanocomposite Gas Sensor for Ethanol Detection. *Journal of Materials Science: Materials in Electronics* 2022, *33* (14), 11498–11510. https://doi.org/10.1007/s10854-022-08124-w.

(6) Makuła, P.; Pacia, M.; Macyk, W. How To Correctly Determine the Band Gap Energy of Modified Semiconductor Photocatalysts Based on UV-Vis Spectra. *Journal of Physical Chemistry Letters*. American Chemical Society December 6, 2018, pp 6814–6817. https://doi.org/10.1021/acs.jpclett.8b02892.
